# Phase-Shift Dynamics of Sea Urchin Overgrazing on Nutrified Reefs

**DOI:** 10.1371/journal.pone.0168333

**Published:** 2016-12-28

**Authors:** Nina Kriegisch, Simon Reeves, Craig R. Johnson, Scott D. Ling

**Affiliations:** Institute for Marine and Antarctic Studies, University of Tasmania, Battery Point, Tasmania, Australia; The University of Hong Kong, HONG KONG

## Abstract

Shifts from productive kelp beds to impoverished sea urchin barrens occur globally and represent a wholesale change to the ecology of sub-tidal temperate reefs. Although the theory of shifts between alternative stable states is well advanced, there are few field studies detailing the dynamics of these kinds of transitions. In this study, sea urchin herbivory (a ‘top-down’ driver of ecosystems) was manipulated over 12 months to estimate (1) the sea urchin density at which kelp beds collapse to sea urchin barrens, and (2) the minimum sea urchin density required to maintain urchin barrens on experimental reefs in the urbanised Port Phillip Bay, Australia. In parallel, the role of one of the ‘bottom-up’ drivers of ecosystem structure was examined by (3) manipulating local nutrient levels and thus attempting to alter primary production on the experimental reefs. It was found that densities of 8 or more urchins m^-2^ (≥ 427 g m^-2^ biomass) lead to complete overgrazing of kelp beds while kelp bed recovery occurred when densities were reduced to ≤ 4 urchins m^-2^ (≤ 213 g m^-2^ biomass). This experiment provided further insight into the dynamics of transition between urchin barrens and kelp beds by exploring possible tipping-points which in this system can be found between 4 and 8 urchins m^-2^ (213 and 427 g m^-2^ respectively). Local enhancement of nutrient loading did not change the urchin density required for overgrazing or kelp bed recovery, as algal growth was not affected by nutrient enhancement.

## Introduction

The opposing forces of herbivory and primary production play vital roles in shaping ecosystems. It is important, yet challenging, to reveal how combinations of these so-called top-down ‘consumption’ and bottom-up ‘resource productivity’ controls operate to ultimately drive shifts in ecosystems from one configuration to another [1,2]. The effects of these two controlling forces can be complex, even in relatively simple ecosystems, as either can have an overriding effect in determining their structure. The net effects of bottom-up and top-down forces can also result in a change in system configuration when an ecosystem is pushed beyond critical thresholds, whereby it may shift into a different state that is stable under environmental conditions identical to the original. In this type of discontinuous ecological transition, or ‘catastrophic’ phase-shift, the system does not return to its former state once conditions are restored to those prior to the shift, and may not easily shift back under most conditions [3–6]. This type of shift has been implicated in the dynamics of ecosystem collapse in terrestrial [7–9], limnetic [10,11], and marine environments ([12–15], reviewed by [16]), and is also considered to pose a dire threat for global ecosystems [17].

For temperate reef environments, the best studied shift between alternative states occurs when rocky reef ecosystems undergo a phase-shift from productive kelp beds to impoverished sea urchin ‘barrens’ [18–21]. While the phenomenon is well recognised, the quantitative nature of local phase-shift dynamics and underlying resilience of kelp beds is in general poorly understood, as are the particular factors that may counter the potential of urchin grazing so that the risk of catastrophic shifts is reduced (reviewed by [22]). Bottom-up forces like nutrient availability are key factors that have the potential to alter the resilience of marine ecosystems [23,24], yet few studies have examined these in concert with grazing pressure (but see [25–27] and none have explicitly manipulated alternative collapse and recovery pathways of phase-shift under different nutrient conditions to quantify the tipping points.

Although numerous studies have demonstrated the impact of urchin grazing on kelp beds (see [22] for overview), most studies focussing on the transition between states have only examined the shift from barrens to kelp beds following removal of urchins [20,28–32]. Fewer studies have assessed the shift from kelp beds to barrens [33,34] and only rarely have the transitions in either direction been addressed concurrently [35,36]. Therefore within particular reef systems there remains a distinct lack of understanding of the range of urchin densities (or biomass density) necessary to precipitate shifts in either direction (reviewed by [22]), and of quantitative estimates of the tipping points.

The question of the potential for interaction among urchin density and nutrient levels is important because along many urbanised coasts phase-shifts resulting in loss of kelp beds is commonly reported [19,36]. In some cases it has been shown that stressors, such as elevated nutrients derived from urbanisation (e.g. [37]), have a detrimental effect on kelp beds by enabling subordinate understory algal species to dominate. Conversely, the so-called ‘stressor’ of enhanced nutrients may act to stimulate primary production to offset the trophic impact of urchin grazing and thus increase resilience of kelp-based systems.

Here, the nature of the transition between kelp beds and urchin barrens in both directions across a range of urchin densities is assessed experimentally, as is the influence on these transitions of adding nutrients to the water column to facilitate kelp growth (i.e. simulating a local increase in a potential stressor often characteristic of near-shore urbanised coastal systems). Specifically, the following questions are addressed: (1) What sea urchin density causes overgrazing of kelp beds?; (2) What urchin density is required to maintain existing urchin barrens?; and (3) Does elevating nutrient levels change the underlying phase-shift dynamics between kelp beds and sea urchin barrens?

## Materials and Methods

### Study area

Our study was conducted at Williamstown Beach in Port Phillip Bay (PPB), Victoria, south-eastern Australia where kelp beds (dominated by *Ecklonia radiata*; (C. Agardh) J. Agardh 1848, Family: *Lessoniaceae*) are declining [38] and remnant kelp beds are under risk of transition to sea urchin barrens characterised by a lack of kelp and other macroalgae. The sea urchin *Heliocidaris erythrogramma* (Valenciennes, 1846; Family: Echinometridae) represents the most wide-spread and ecologically important herbivore in PPB and demonstrates active overgrazing of kelp beds [39,40]. *H*. *erythrogramma* occurs throughout PPB and at local scales can occur at high densities (up to ~ 120 individuals m^-2^).

To facilitate manipulating sea urchin densities and administering elevated nutrient conditions, ‘artificial’ patch reefs were constructed from naturally occurring boulders which were placed on sand substrata adjacent to Williamstown Beach (S 37° 52’ 10.5564”, E 144° 53’ 36.4884”). The site is characterized by shallow sub-tidal rocky reef with large boulders and cobbles interspersed by sand patches. The majority of the sub-tidal reef area (2 to 5.5 m depth) is *H*. *erythrogramma* sea urchin barrens, but a small number of remnant kelp patches dominated by *E*. *radiata* can be found. The macroalgal community consists of up to 95% cover of *E*. *radiata*, the fucoid *Sargassum vestitum* ((R. Brown ex Turner) C. Agardh 1820, Family: Sargassaceae) and the introduced Japanese kelp *Undaria pinnatifida* ((Harvey) Suringar, 1873, Family: Alariaceae) with sparse understorey growth, while on the shallowest margin of the sub-tidal area (1.5 m), *Sargassum spp*. are the dominant canopy-forming macroalgae.

In the study area the sea urchin *H*. *erythrogramma* occurred at an average density of 4.6 m^-2^ in local urchin barrens and 5.2 m^-2^ in kelp beds (at a biomass density of 245 g m^-2^ and 539 g m^-2^ respectively). Following pilot trials at this site over 6 months, a large sand patch (2,040 m^2^) surrounded by rocky reef in a depth of 4 m was selected as a suitable habitat upon which to build experimental boulder patch reefs. Pilot trials demonstrated that natural sand barriers were effective in limiting migration of urchins between patch reefs, enabling urchin density treatments to be maintained efficiently. This approach was superior to using artificial fences or cages, which quickly became fouled and required weekly maintenance.

### Experimental design and data collection

#### Experimental patch reefs

Using SCUBA, small boulders and cobbles (0.20–0.50 m diam.) were sourced from the adjacent rocky reef and constructed into 28 patch reefs (each ~ 0.85 m^2^ planar area) on the sand patch. Reefs were separated by at least 5 m from each other and from the surrounding natural reef. This distance was chosen *a priori* to achieve independence between replicate reefs in respect to the nutrient enhancement treatments applied in the experiment (after [23,41] and also confirmed for this study, see ‘Nutrients’ below). All boulders and cobbles used to construct the patch reefs were scraped clean of foliose macroalgae (except encrusting red algae) and sessile invertebrates before commencing the experiment. A star picket was driven into the substratum in the centre of each reef to provide a reference point for assessments and as a fixture to hold bags containing nutrient. An additional star picket was positioned next to each reef for the purpose of holding an ‘onion bag’ containing two reproductive *E*. *radiata* individuals to ensure that *E*. *radiata* propagules were not a limiting factor for kelp re-establishment across all experimental reefs. The bags were kept clean from epiphytes and *E*. *radiata* bearing sori continued to grow within the bags, and were exchanged at least every 2 months. The 28 boulder reefs were assigned randomly to a total of 4 treatments representing combinations of ‘reef ecosystem state’ (kelp beds *vs* urchin barrens) and ‘nutrient’ conditions (ambient *vs* enhanced), and within each treatment the 7 replicate reefs supported urchins at densities of 0, 4, 8, 12, 15, 20 and 24 urchins m^-2^.

#### Reef ecosystem state

Three adult *E*. *radiata* individuals were transplanted to each appropriate patch reef to simulate the ‘kelp bed state’, providing a canopy cover of ~ 50% within the internal 0.25 m^2^ of each patch reef. Kelp attached to small boulders were collected from the nearest kelp bed and first placed on the edge of the sand patch to acclimatise. They were left there for 1 week to eliminate any potential dislodgement or damage due to handling before placing them on the prepared boulder reefs. The ‘barrens state’ consisted of cleaned boulders without the addition of kelp.

#### Nutrients

A mesh bag (mesh size 1 x 1 mm) with 200 g of slow release fertiliser (Osmocote^®^ Pro 3–4 M, 17N:4.8P:8.3K) was used to enhance nutrients on individual patch reefs (quantity of fertiliser and technique after [41]). Patch reefs with no nutrient enhancement were assigned a mesh bag with small pebbles as procedural control. Mesh bags with fertiliser were exchanged every 6 weeks to ensure constant supply of nutrients. To confirm that nutrient concentrations were significantly enhanced using this procedure, water samples were taken from both ‘elevated nutrient’ and ‘control’ patch reefs midway through the period of routine fertiliser application (i.e. 3 weeks after application fertiliser). Samples were taken 30 cm from the nutrient bag with a 60 ml plastic syringe, then filtered and placed on ice and analysed at the Water Studies Centre (School of Chemistry, Monash University, Victoria) for concentrations of nitrogen-oxides, ammonia and filterable reactive phosphorus (Table 1). Nutrient concentrations across ‘ambient’ *vs*. ‘nutrient enhanced’ reefs were compared using one-way analysis of variance (ANOVA), with 3 replicates for each level. Analysis showed significant enhancement of concentrations of ammonia and all nutrients combined (1-way ANOVA, *F*_1,4_ = 10.76, P < 0.05 and *F*_1,4_ = 18.86, *P* = 0.01 respectively; Table 1).

**Table 1:** Mean values and results of 1-way ANOVAs of nutrient concentrations for ‘Ambient’ and ‘Enhanced’ nutrient conditions on experimental patch reefs.

	Ambient	Enhanced	F	*P*
**NH**_**3**_	**0.27 (± 0.07)**	**0.69 (± 0.1)**	**10.76**	**< 0.05**
**NO**_**x**_	0.04 (± 0.01)	0.06 (± 0.01)	4.00	0.12
**FRP**	1.56 (± 0.03)	1.66 (± 0.07)	1.82	0.25
**Total**	**1.88 (± 0.06)**	**2.41 (± 0.11)**	**18.86**	**0.01**

Mean values (μmol l^-1^ ± SE, n = 3) and results of ANOVAs are shown for ammonia (NH_3_), nitrate and nitrite combined (NO_x_), filterable reactive phosphorus (FRP) and the sum of all nutrients (Total). Values in bold are significant at α = 0.05. The transformation of Y^-9^ was used for FRP, none of the other concentrations needed a transformation.

#### Sea urchins

After randomly assigning the 4 different ‘reef state’ and ‘nutrient condition’ treatments to the experimental patch reefs, each of the 7 replicate patch-reefs within each treatment received (at random) one of 7 different urchin densities (0, 4, 8, 12, 15, 20 and 24 urchins m^-2^). Reefs were then checked every 3 weeks for the 12 months of the experiment to ensure urchin densities were maintained at their designated level. The range of urchin densities selected was based on surveys conducted on reefs across northern PPB, which showed mean urchin densities of 4.6 ± 0.5 urchins m^-2^ (245 ± 45 g m^-2^, n = 25 random 1 x 1 m^2^ quadrats) and 5.2 ± 0.7 urchins m^-2^ (539 ± 80 g m^-2^, n = 25 random 1x1 m^2^ quadrats) for sea urchin barrens and kelp beds respectively. Thus, urchin densities chosen for the experiment ranged between 0 to ~5 times the mean observed densities for barrens and 0 to 4.5 times the mean for reefs starting as the ‘kelp bed’ state.

#### Assessments

The experiment was run for ~13 months, from November 2012 to December 2013, and the amount of erect macroalgae on each reef was assessed every 3 months. Assessments were undertaken on SCUBA with *in situ* estimates of percentage cover of canopy-formers and understorey. Percentage cover assessments were performed using the point-intercept method (a 50 by 50 cm quadrat with 49 regularly spaced points defined by intersecting string lines, and an additional random point within the quadrat assigned in advance). Stipe counts were used to enumerate the abundance of canopy-forming brown algae (*E*. *radiata*, *S*. *vestitum*, and the introduced ephemeral Japanese kelp *U*. *pinnatifida*), and on the final assessment the total number of macroalgal species on each patch reef was counted.

#### Statistical analysis

The effect of ‘reef state’ and ‘nutrient conditions’ on canopy-forming algae was assessed across the gradient of sea urchin densities. The analyses were undertaken in two parts; initially the rate of shift between reef states (in both directions) was investigated by analysing the planar % cover of canopy-forming algae with a 1-way Model I Analysis of Covariance (ANCOVA) using the factor ‘nutrients’ (2 levels: ambient *vs*. enhanced) and with sea urchin density as the covariate. Second, macroalgal species richness and diversity (Shannon-Wiener Index) at the end of the experiment was calculated and analysed using a 2-way Model I Analysis of Covariance (ANCOVA) with factors ‘reef state’ (2 levels: kelp *vs*. barrens) and ‘nutrients’ (2 levels: ambient *vs*. enhanced), with sea urchin density as the covariate. Because the data contained some zeros due to complete overgrazing or lack of kelp recovery, the assumption of homoscedasticity was violated (slightly) in some cases even after transformation of the data, but since the design was balanced the impact of the violation on error rates and power is unlikely to be problematic. The transformation to stabilise variances was determined using the *Box-Cox* procedure. *RStudio* (Version 0.98.953—© 2009–2013 *RStudio*, *Inc*.) was used for all statistical analyses.

## Results

### Kelp bed overgrazing

**Fig 1:**
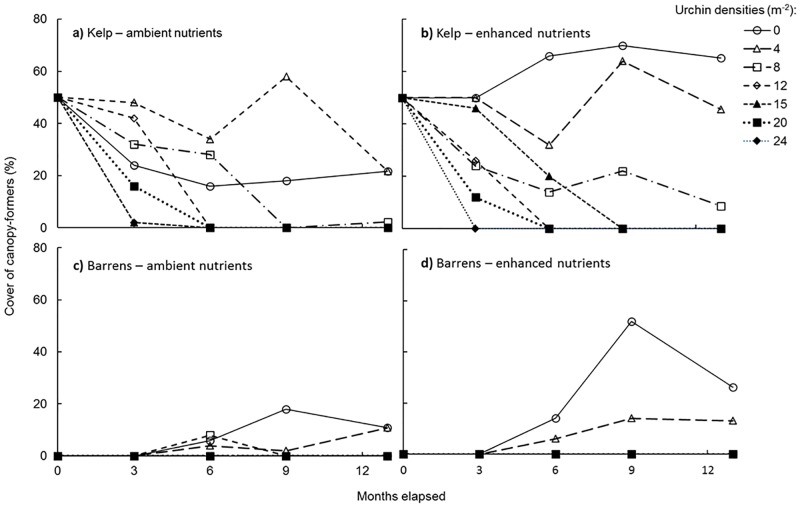
Kelp collapse and recovery at different urchin densities over time. Percentage cover of canopy-forming algae at a range of sea urchin densities (individuals m^-^²) on patch reefs initiated as the ‘kelp bed’ (a) and (b), and ‘barrens’ state (c) and (d) in northern Port Phillip Bay, Nov. 2012 to Dec. 2013. Plots (a) and (c) represent reefs with ambient nutrient levels, and (b) and (d) show results for reefs with enhanced nutrient levels.

Reefs initiated in the ‘kelp bed’ state demonstrated rapid decline in kelp cover when subject to high densities of grazing urchins (Fig 1a and 1b). The higher the sea urchin density, the more rapid the loss of kelp (Fig 1a and 1b). Decline in kelp cover under moderate to high urchin densities (12–24 urchins m^-2^) occurred within 3 months (Fig 1a and 1b; Table 2). After 13 months, any patch reef initiated as a ‘kelp bed’ exposed to urchin densities of ≥ 8 m^-2^ (an equivalent urchin biomass density ≥ 427 g m^-2^) was completely denuded of kelp. Conversely, intact kelp remained on reefs with ≤ 4 urchins m^-2^ (an equivalent biomass density ≤ 213 g m^-2^) (Fig 1a and 1b). This clear response of kelp cover to sea urchin density was independent of nutrient levels (Table 2).

**Table 2:** Results of 1-way fixed effects model I Analysis of Covariance testing the differences in cover of canopy-forming macroalgae at different urchin densities (covariate) and nutrient conditions (ambient *vs*. enhanced) for the 4 periods of *a priori* interest across a quarter (3 months), half (6 months), three quarters (9 months) and a full year (13 months).

	3 months				6 months				9 months				13 months			
	df	MS	F	*P*	df	MS	F	*P*	df	MS	F	*P*	df	MS	F	*P*
**a) Overgrazing**																
Interaction term	1	43.70	0.23	0.64	1	0.008	0.006	0.94	1	0.16	0.19	0.68	1	0.01	0.03	0.88
Urchins (cov)	1	2409.9	13.83	**< 0.01**	1	26.26	22.56	**< 0.001**	1	19.50	24.60	**< 0.001**	1	27.65	39.51	**<0.001**
Nutrients	1	126.00	0.72	0.41	1	0.98	0.84	0.38	1	0.67	0.85	0.38	1	0.60	0.86	0.37
Residuals	11	174.27			11	1.16			11	0.79			11	0.70		
**b) Recovery**																
Interaction term	-	-	-	-	1	14.00	1.68	0.22	1	284.46	2.33	0.15	1	44.34	1.48	0.25
Urchins (cov)	**-**	**-**	-	**-**	1	115.35	13.05	**<0.01**	1	14.52	16.39	**< 0.01**	1	456.23	14.57	**<0.01**
Nutrients	**-**	**-**	-	-	1	7.14	0.81	0.39	1	0.50	0.56	0.47	1	21.60	0.69	0.42
Residuals	**-**	**-**			11	8.84			11	0.89			11	31.33		

The interaction term (nutrients*urchins) is the test for homogeneity of slopes. (a) Results of reefs starting as the ‘kelp bed state’, and (b) results of reefs starting as the ‘urchin barrens state’. Note that no recovery of canopy-formers was observed after 3 months at any urchin density. Values in bold are significant at α = 0.05. Transformations used for overgrazing (a) were ln(Y) for the assessment at 6 months, and Y^-0.5^ for the assessment at 9 months, while no transformations were used for data collected at 3 and 13 months. For the recovery (b), data were transformed as ln(Y) for the assessment at 9 months, while a transformation was not required for the other assessments.

### Kelp bed recovery

**Fig 2:**
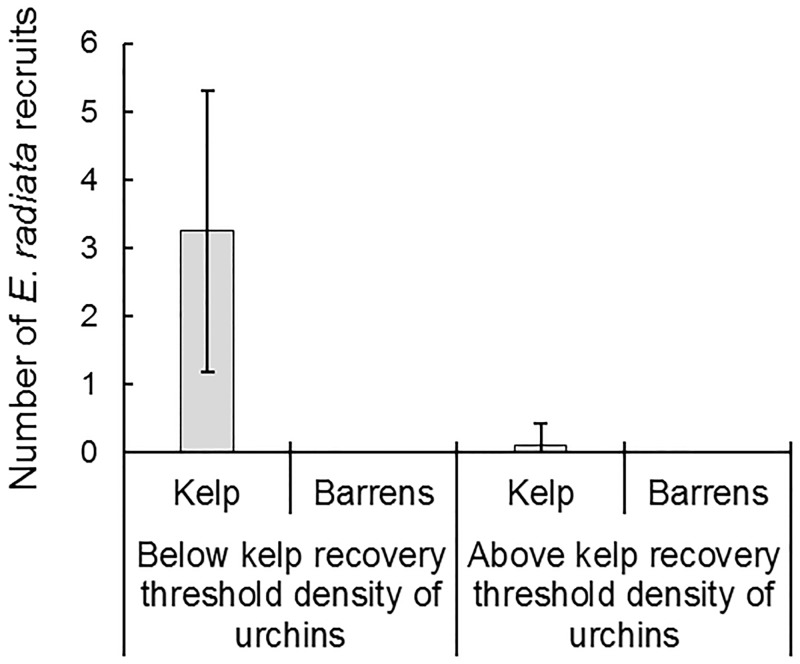
*E*. *radiata* recruitment. Abundance (mean ± SE) of *E*. *radiata* recruits on reefs above and below the critical urchin density (4 urchins m^-2^) for kelp recovery after 13 months (‘Kelp’ and ‘Barrens’ refers to the initial states of reefs). Nutrient enhancement did not influence recruitment and therefore data for ‘nutrient enhanced’ and ‘ambient nutrient’ reefs were pooled for display.

On patch reefs initiated as urchin barrens, recovery of kelp after 6 months was only observed for sea urchin densities ≤ 4 m^-2^ (equivalent biomass density ≤ 213 g m^-2^). Kelp cover increased on reefs with low urchin densities, however no kelp growth was observed on reefs with urchin densities ≥ 8 m^-2^ (equivalent biomass density of ≥ 427 g m^-2^) (Fig 1c and 1d). Analysis of the effects of ‘reef ecosystem state’ and ‘nutrients’ on the response of kelp cover over the range of sea urchin densities examined showed clearly that the only factor influencing regrowth of kelp canopy was sea urchin density, with nutrient levels having no detectable effect on canopy cover (Table 2). After 13 months, canopy species recovering on reefs starting as the ‘barrens state’ were *S*. *vestitum* and *U*. *pinnatifida*. On reefs starting in the ‘kelp bed state’, 14 *E*. *radiata* recruits were found after 13 months, with 13 of these kelp recruits observed on reefs where adult *E*. *radiata* were still present at the end of the experiment (i.e. on reefs that were below the ‘recovery threshold urchin density’ of 4 urchins m^-2^, Fig 2). Only a single recruit was found on a reef which started in the ‘kelp bed state’, but which supported urchins at a density above this threshold. Six kelp recruits were found on nutrient enhanced reefs and 8 on reefs experiencing ambient nutrient conditions.

### Algal community response

**Fig 3:**
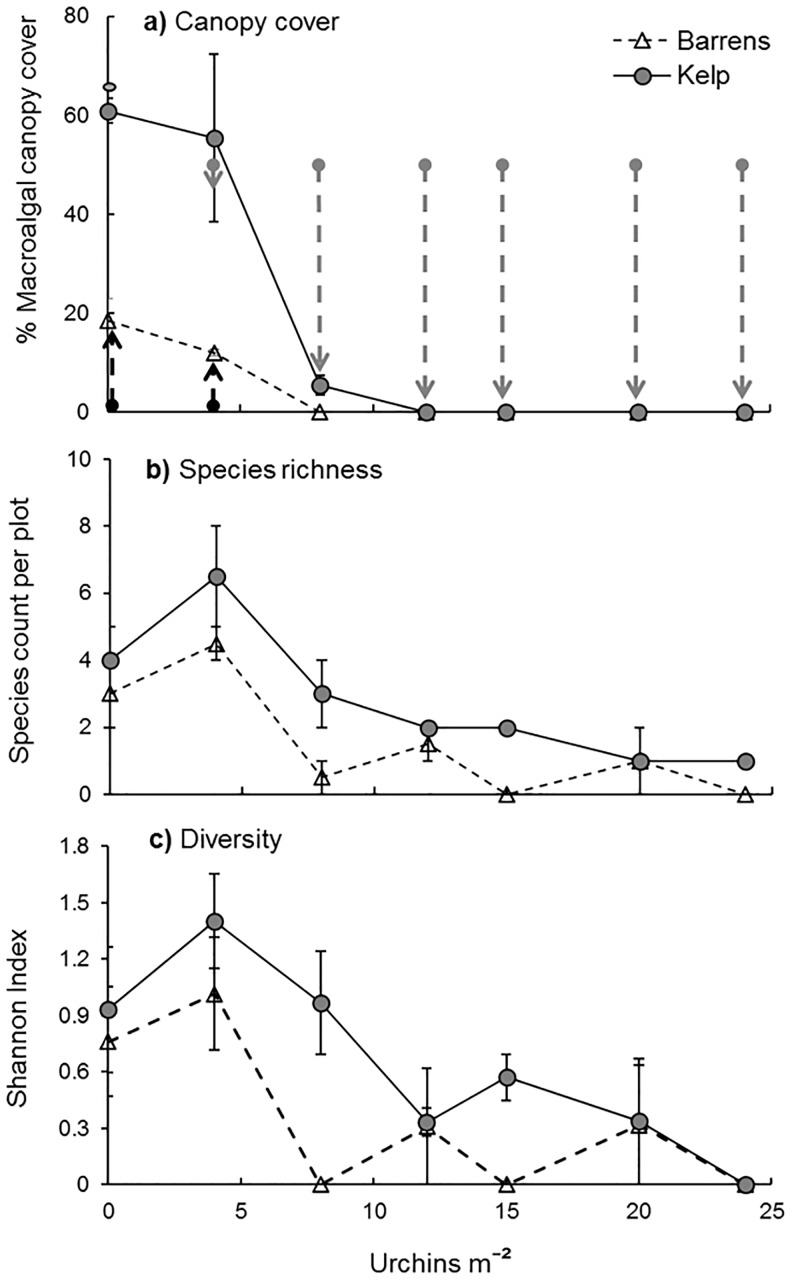
Dependence of algal species richness and diversity on canopy cover. Panel a) details cover of canopy-forming algae, b) shows macroalgal species richness, and c) macroalgal species diversity, against sea urchin densities at the end of the 13 month experimental period. Reefs with initial states of ‘kelp bed’ and ‘urchin’ barrens’ are indicated by a grey circle and a white triangle respectively; data are means ± SE. Reefs with enhanced nutrients have been pooled with reefs with ambient nutrient conditions since the addition of fertiliser did not influence response variables (see Tables 2 and 3). Note that species present at the start of the experiment (*E*. *radiata* for kelp bed reefs and encrusting red algae for all reefs) were excluded from the analysis. Arrows in (a) show responses to experimental manipulation of sea urchin biomass in kelp beds (thick grey arrows = forward-shift ‘collapse’ from kelp to urchin barrens) and on sea urchin barrens (thin black arrows = reverse-shift ‘recovery’ from urchin barrens back to kelp beds).

Examination of the community composition of fleshy algae growing on experimental patch reefs at 13 months (2-way fixed effects ANOVA on species richness and Shannon Diversity) revealed no overall effect of nutrients, while the starting state of the reef and sea urchin density significantly influenced algal cover (Fig 3b and 3c, Table 2). Increasing sea urchin densities resulted in declining macroalgal species richness and diversity while, unsurprisingly, reef patches initiated in the ‘kelp bed state’ showed higher algal species richness and diversity. Yet, as was evident for *E*. *radiata*, a precipitous decline in species richness occurred at urchin densities between 4 and 8 m^-2^ (Fig 3a and 3b). Interestingly, algal species richness as well as Shannon diversity was highest at 4 as opposed to 0 urchins m^-^². The impoverished algal community on reefs with ≥12 urchins m^-2^ chiefly consisted of the early colonising species *Ulva sp*. and the ephemeral *Stenogramme interrupta* ((C. Agardh) Montagne) which rapidly monopolize available space when grazing pressure is low.

**Table 3:** Results of 2-way fixed effects model I Analysis of Covariance testing the significance of differences in responses of species richness (a) and Shannon diversity (b) across different urchin densities (covariate), dependent on initial ‘reef state’ (kelp *vs*. barrens) and nutrient conditions (ambient *vs*. enhanced) after 13 months of treatment.

	df	MS	F	*P*
**a) Species count**				
Interactions with covariate	1, 20	0.29	1.58	0.23
Urchins (cov)	1	5.1	29.64	**< 0.001**
Reef state	1	1.63	9.50	**< 0.01**
Nutrients	1	0.006	0.04	0.85
Reef state x Nutrients	1	0.27	1.54	0.23
Residuals	23	0.17		
**b) Shannon diversity (H’)**				
Interactions with covariate	1, 20	0.03	0.53	0.67
Urchins (cov)	1	1.37	23.01	**< 0.001**
Reef state	1	0.37	3.19	**< 0.05**
Nutrients	1	0.00	< 0.001	0.98
Reef state x Nutrients	1	0.07	1.22	0.28
Residuals	23	0.06		

The combined interactions with the covariate (urchins * reef state + urchins * nutrients + urchins * reef state * nutrients) tests for homogeneity of slopes. Values in bold are significant at α = 0.05. Transformations used were *Y* ^0.45^ for species richness (a) and *Y* ^0.5^ for Shannon diversity (H).

## Discussion

### Tipping-points of kelp bed collapse and recovery

In this study the density of the sea urchin *H*. *erythrogramma* required to overgraze kelp on experimental patch reefs was ≥ 8 urchins m^-2^ (equivalent urchin biomass density of 427 g m^-2^). In contrast, kelp recovery on patch reefs initially starting as ‘barrens’ occurred at urchin densities ≤ 4 m^-2^ (equivalent biomass density of 213 g m^-2^). These observations might be interpreted as the existence of a single tipping point between 4 and 8 urchins m^-2^. However, consistent with other observations worldwide [42] it is more probable that there are different tipping-points in *H*. *erythrogramma* density that either drive destructive overgrazing or enable kelp bed recovery, and thus indicate a hysteresis in this system (reviewed by [22,43]). The hysteresis would then be found in between 4 and 8 urchins m^-2^ (equivalent to 213 and 427 g m^-2^ urchin biomass density respectively) with recovery closer to 4 and overgrazing closer to 8 urchins m^-2^.

Many previous studies have demonstrated that sea urchins cause overgrazing and that kelp bed recovery is possible by simply removing urchins from the system (e.g. [21,39,44]). However, in these ‘all or nothing’ studies, gradients in the density of sea urchins are not explored and typically only a single initial state (i.e. urchin barrens) is considered so that tipping-points that precipitate phase-shift in either direction are elusive. Few studies have ever examined both the response of kelp beds and urchin barrens to gradients in sea urchin density [35,36,45,46]. To date, Hill, Blount et al. [35] performed the most detailed experiments on density-dependent urchin overgrazing by separating ‘collapse’ and ‘recovery’ shifts. However, they did not estimate transition thresholds and the recovery and overgrazing experiments were conducted as separate experiments at marginally different scales, in different locations and using different urchin densities across ‘kelp bed’ and ‘barrens’ reef states, so it is difficult to identify hysteresis from their experiments.

Additionally, most studies using sea urchin manipulations report sea urchin density, but not the biomass required to shift from kelp to barrens or *vice versa*. While Filbee-Dexter and Scheibling [43] and Ling, Scheibling et al. [22] both give estimates of densities and biomasses for tipping-points derived from studies worldwide, there remains a lack of definitive studies within particular systems that determine local phase-shift dynamics. It is important to acknowledge that urchins of the same species not only occur at highly variable densities across different sites, but their size structure and thus biomass can also differ substantially among sites [22]. Only by considering biomass density (g m^-2^) can variability in the abundance and size of herbivores be taken into account to estimate tipping-points across grazer-driven systems, thus enabling meaningful comparison between different study areas, times and species. Furthermore, it is important to note that the mean urchin biomass measured within remnant kelp beds in northern PPB was ~ 539 g m^-2^ which is 112 g greater than the experimentally estimated tipping-point in urchin biomass of kelp bed overgrazing. This observation is consistent with the observed steady decline of remnant beds in this region from 2009 to 2015 (Authors pers. obs.; S. Reeves, unpublished data).

### Kelp bed recovery

**Fig 4:**
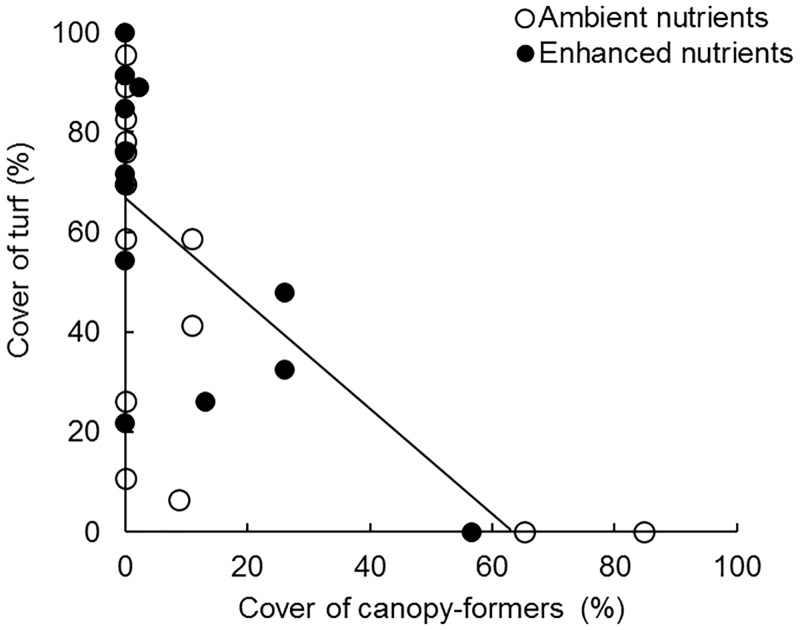
Percentage cover of turf-forming algae *versus* cover of canopy-formers. Open circles display reefs with ambient nutrient conditions and filled circles are reefs with enhanced nutrient levels across all experimental patch reefs at the conclusion of the 13 months experiment (the treatment of ‘reef state’ is not indicated). Fitted line represents treatments pooled across ambient and enhanced nutrient reefs (*R*² = 0.75, y = -0.056x + 4.16, values derived from linear regression with transformation ln(Y)) because a 1-way ANCOVA showed no significant difference in relationships between reefs with enhanced nutrients and those with ambient nutrient levels (homogeneity of slopes: *F*_1,24_ = 0.85, *P* = 0.36; test between treatments after factoring for the covariate, *F*_1,25_ = 1.00, *P* = 0.33).

While kelp was removed relatively quickly at sea urchin densities ≥ 8 m^-2^, in the treatments where it occurred, the rate of kelp recovery was comparatively slow over the 13 month duration of the experiment. Reductions in urchin densities (usually complete urchin removal) have been shown to be effective in rehabilitating kelp beds in manipulative and ‘natural’ experiments [20,44,47,48], however by considering a range of urchin densities this experiment explored possible thresholds of urchin density to allow recruitment and growth of kelp *E*. *radiata*. Importantly, recruitment was notably greater in the presence of adult sporophytes. This is contrary to the studies of Carnell and Keough [49] and Flukes, Johnson and Wright [50], who showed greater recruitment of *E*. *radiata* into patches cleared of canopy-forming algae. There are 2 principal mechanisms that might account for the divergent results among the difference studies. The first addresses the effects of the scale of the kelp bed patch and extent of intraspecific competition. Carnell and Keough [49] and Flukes, Johnson and Wright [50] worked in large beds of dense kelp where the benthos was heavily shaded (>100% canopy cover) and there was (ostensibly) an abundance of spore production. In contrast, since the small patch reefs initiated in the ‘kelp bed state’ supported only 3 adult sporophytes defining 50% canopy cover, light levels on these patches were likely higher than on larger kelp patches with a similar density of individuals (C. Layton, unpublished data), and thus the effects of intraspecific competition are likely much reduced relative to larger kelp bed patches. Secondly, it is likely that there is an interaction between the timing of the availability of substratum to which new sporophytes can recruit, and the effect of adult kelps sweeping the substratum in surge. Kennelly [51] showed that timing of canopy clearance has a large influence on recruitment of *E*. *radiata*, and both Carnell and Keough [49] and Flukes, Johnson and Wright [50] cleared patches within established kelp canopies just before the seasonal peak (during autumn) in *E*. *radiata* sorus development and spore release. Studies with other kelps have also clearly demonstrated that the timing of canopy disturbance relative to seasonal availability of spores is a major determinant of recruitment (e.g. [48,52–54]). In the current study, experimental reefs were constructed in late spring and therefore were overgrown by understorey algae before *E*. *radiata* was reproductive, which is likely to have affected the ability of zoospores to settle and gametophytes and sporophytes to develop and gain a foothold on the reef [51]. Given the higher rate of kelp recruitment on patch reefs supporting adult sporophytes compared to those devoid of kelp, it appears that the presence of adult *E*. *radiata* can enhance recruitment not only through supplying a local source of propagules (which was controlled across all experimental reefs), but also by influencing the surrounding benthos. A likely mechanism is that the sweeping action of *E*. *radiata* fronds on the substratum as a result of water motion [55,56] reduces the abundance of other algae (particularly turf-forming species) that compete with kelp sporelings or gametophytes. In the current experiment cover of turf-forming algae on experimental patch reefs declined with increasing cover of adult *E*. *radiata* sporophytes (Fig 4). Overall, these results suggest that rehabilitation of kelp beds on urchin barrens will be most readily facilitated by actively transplanting adult kelp, once urchins have been reduced to densities below the recovery threshold. The adult sporophytes therefore appear to play a dual role by providing both propagules and also reducing the cover of competing understorey in the form of turfing algae.

### Nutrient enhancement

Nutrient measurements for the current study site throughout 2013 showed an average nitrogen concentration (nitrite, nitrate and ammonia) of 0.76 μmol l^-1^ (± 0.11), which suggests nitrogen may be limiting algal growth [57,58]. This concentration is lower than that reported for other temperate reef systems (e.g. North-West America, [59], South-West Finland, [60]), slightly higher than published levels for western Australia, and below measurements from New South Wales [23]. Given this, it was expected that nutrient enhancement would have a strong effect on algal growth. However nutrient enhancement did not have any detectable effects on either rates of kelp loss or recovery on the experimental reefs. Seeing that the concentrations of ammonia were significantly higher on reefs with enhanced nutrients but neither phosphorus nor nitrogen-oxides showed higher concentration suggests that at least ammonia was enhanced sufficiently to promote algal growth if this was a limiting factor for production. The technique of nutrient addition was in accordance with other experiments and even exceeded the amount which was added to experimental units in those studies [23,26,41], so the fact that elevated nitrogen-oxide concentrations were not detected on ‘nutrient enhanced’ reefs in the current study suggests rapid uptake by microbes, phytoplankton, and microphytobenthos. PPB experiences high rates of denitrification as a result of metabolism of microorganisms [61] and therefore nitrogen-oxides in the water disappear very quickly. This is evident in the discrepancy in the input of nitrogen and what can be found in the water column [62]. A large sewage treatment plant to the west (i.e. Western Treatment Plant) contributes ~3400 tons of Nitrogen year^-1^ and the Yarra River in the North adds ~ 1050 tons year^-1^ [61], but concentrations of nitrogen are still low in the water column in PPB [62].

### Algal community response

Species richness and species diversity of fleshy algae was observed to be highest at an urchin density of 4 m^-2^ (equivalent to 213 g m^-2^ biomass), which implies that grazing in moderation, or selective grazing on canopy-forming kelp, may release the growth of subordinate understorey algal species leading to a more diverse algal community than in the complete absence of grazing [63]. *H*. *erythrogramma* shows preferential grazing on *E*. *radiata* in eastern Australia (for PPB N. Kriegisch, unpublished data; for NSW see Wright et al. [45] and Hill et al. [35]), suggesting that the cause of increased algal diversity at moderate grazing is the result of increased space for the recruitment and growth of less palatable algal species. Due to this selective grazing, this grazer-driven stimulation of both algal diversity and richness may ultimately increase overall resilience of the ‘kelp bed’ community when kelp production exceeds grazing rates.

## Conclusion

Identifying, defining and anticipating critical transitions in nature remain key challenges for understanding and ultimately managing human impacts on natural ecosystems. Here, for the first time, we have used an experimental approach to explore both collapse and recovery dynamics of kelp bed systems prone to sea urchin overgrazing. Consistent with other research, this approach indicates the existence of multiple tipping-points in this system and thus the discontinuous or ‘catastrophic’ nature of transitions between kelp beds and urchin barrens. This study supports the notion that kelp beds and sea urchin barrens exist as alternative stable states of temperate sub-tidal rocky reefs. Furthermore, we have shown that the dynamics of this phase-shift are unaffected by the local enhancement of nutrients, as no evidence was found that the enhancement had any effect on algal production. Importantly, this study has identified the urchin densities at which the ecosystem tipping-points may be found. This information is key for managing the resilience of desirable ecosystem states and for implementing practical measures to avoid being locked into impoverished low-value ecosystem configurations.

## Supporting Information

S1 Dataset Data used in analyses. (XLSX)
